# VITILIGO AND THE MELANOCYTE RESERVOIR

**DOI:** 10.4103/0019-5154.57604

**Published:** 2009

**Authors:** Rafael Falabella

**Affiliations:** *From the Universidad del Valle, Carrera 38A # 5A-100, Centro Dermatológico de Cali - CDC, Calle 5B3 # 38-44, Cali, Colombia.*

**Keywords:** *Hair follicle*, *melanocyte*, *reservoir*, *stem cells*, *vitiligo*

## Abstract

Repigmentation of vitiligo depends on available melanocytes from three possible sources: from the hair follicle unit which is the main provider of pigment cells, from the border of vitiligo lesions, and from unaffected melanocytes within depigmented areas; pigment cells at these locations originate a perifollicular, border spreading and a diffuse repigmentation pattern. In order for repigmentation to take place under stimulation with diverse therapies, melanocytes should be present in appropriate numbers. Melanocyte tissue stem cells located in the niche at the bulge region of the hair follicle are the most important sources for providing immature pigment cells that undergo terminal differentiation and originate repigmentation, but cytokines, UVR and other molecules acting in melanogenesis with adequate regulation mechanisms contribute to successful recovery in vitiligo. The presence of keratinocyte stem cells in the interfollicular epidermis raises the question on the possibility of melanocyte stem cells in a similar location and the development of future strategies for therapeutic purposes.

## Introduction

In vitiligo, melanocytes are targeted by multiple aggressions leading to marked reduction of pigment cells and eventually to their complete destruction.[1] In some patients, the noxa may be so severe that melanocytes also disappear from the hair follicle units where they act as the most important pigment cell reservoir

Vitiligo recovery depends on a viable melanocyte reservoir and in many patients with vitiligo repigmentation is possible when pigment cells are stimulated with appropriate topical or oral medications. Nevertheless, when pigment cells are completely destroyed, melanocyte transplantation may the best therapeutic approach.[2]

In the following sections, the presence of pigment cells in the hair follicle and their relationship with vitiligo are discussed according to available evidence.

## Definition of the Melanocyte Reservoir

**Figure 1:**
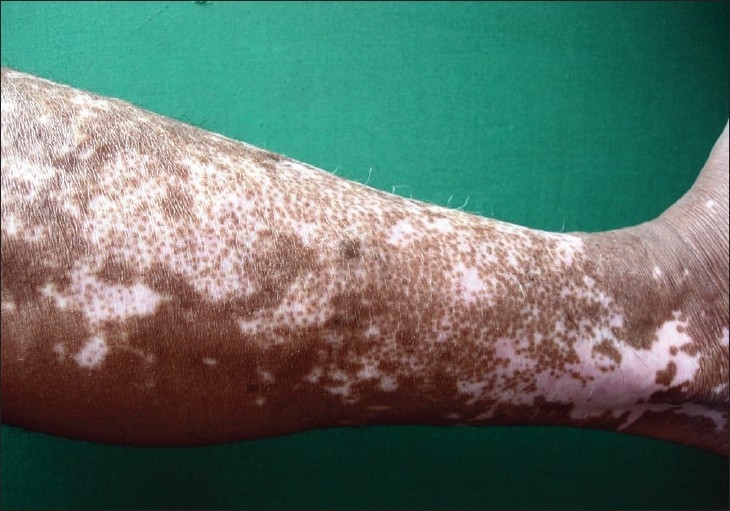
Perifollicular repigmentation pattern. Multiple small pigmented spots around hairs at different stages of progression are observed. In some areas, coalescence of these pigmentary islands leads to complete repigmentation

**Figure 2:**
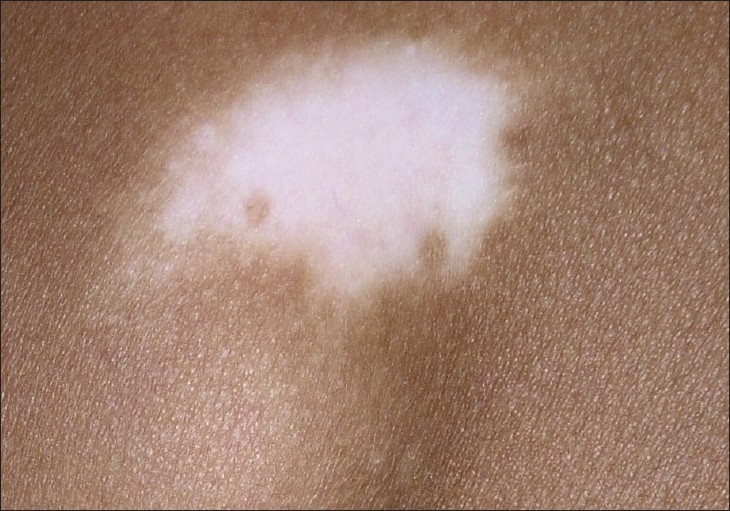
Marginal repigmentation pattern. Repigmentation around the periphery of lesion advancing 4-5 mm from the edge may be observed

**Figure 3:**
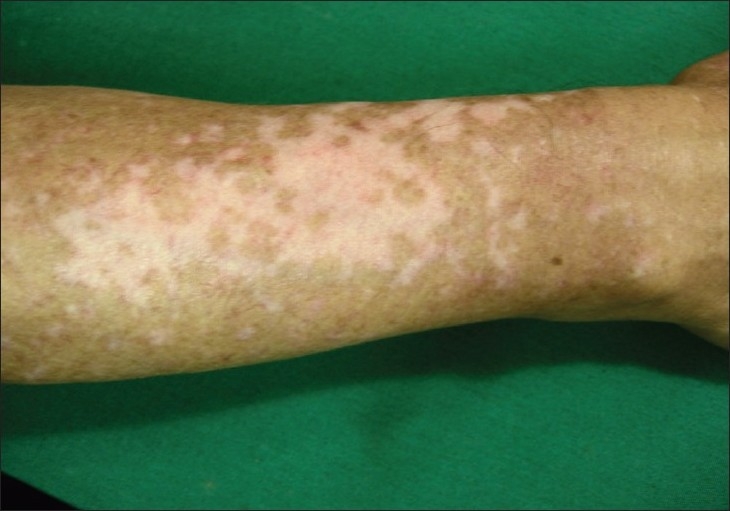
Diffuse repigmentation pattern. Several patches of repigmentation on the dorsum of forearm in this patient indicate recovery with a diffuse pattern

Although the concept of melanocyte reservoir is mostly used to designate the hair follicle unit, melanocytes for repigmentation by medical methods arise from three main sources: (a) the hair follicle unit; (b) unaffected melanocytes within areas of depigmented epidermis, and (c) melanocytes located at the edge of vitiligo lesions.[3] When studying the patterns of repigmentation in 125 patients with 352 vitiligo patches treated with different methods, the following repigmentation patterns were observed: (1) perifollicular when treated with systemic and topical PUVA [Figure 1]; (2) marginal, after systemic PUVA and topical calcipotriol [Figures 2 and 3] diffuse, when on topical steroids [Figure 3].[4] Nevertheless, most melanocytes originate from the hair follicle unit where they are present in large numbers and migrate towards the epidermis. A striking feature of the hair follicle reservoir is the enormous potential for providing pigment cells considering its minute size and diameter.

It is of special importance to emphasize that hair follicles are absent on palms, soles, mucosal or semi-mucosal surfaces and therefore these areas become particularly refractory to all therapies because the melanocyte reservoir is lacking; however, a diffuse repigmentation pattern is frequently observed,[4] raising the question of an additional reservoir in the rete pegs outside the hair follicle.

## Historical Aspects

Vitiligo is a disease that was observed very early in history, and most ancient civilizations and religions had some type of reference about lack of pigmentation.[5] One of the earliest terms was “Kilas” in the Rig Veda, which was meant as similar to a white spotted deer.[6] The Ebers Papyrus in 1550 BC mentioned two forms of depigmentation that could be interpreted as leprosy or depigmentation resembling vitiligo.[7] By 1400 BC white leprosy spots were called Sveta khushtha in the Atharva Veda[8] and in 1200 BC Japanese Shinto prayers described depigmentation in the Amarakosa.[5,7] Around 600 BC, the Ashtanaga hridaya explained prognostic factors of depigmentation.[7] In 250 BC, Ptolemy II translated the Bible from Hebrew into Greek and in the Leviticus XIII (Old Testament), the word Zara'at used for different skin conditions was translated as “lepros” (scales) that was misinterpreted later on as leprosy and other hypopigmented disorders defined as unclean diseases.[5,9,10] In 200 BC, the Indian Manu Smriti described “Sweta Kushtha” meaning “white disease” probably referring to vitiligo.[8] Herodotus (484-425), the Greek historian, claimed that foreigners ‘had sinned against the sun’ and must leave the country.[9] Years later, the term vitiligo was perhaps derived from the latin word vitelius[10] and used to describe the white flesh of calves, and finally the word vitiligo was attributed to Celsus in his classic Latin book De Medicina in the first Century AD.[5,11]

Many centuries went by and vitiligo continued to be one of the most important depigmentation ailments worldwide provoking discrimination or segregation in certain cultures, where affected individuals were unable to get jobs or even become married[12] most probably based upon ancient religious beliefs.

After the middle ages, around 1533 Andreas Vesalius called the attention about the skin having two layers.[13] Several decades later, Jean Riolan the Younger (1580-1657) separated the skin of a black subject into the upper black layer (horny layer) and the lower white layer “as snow” (dermis).[14] In 1665, Marcello Malpighi proposed that skin colour was mainly determined by the granules of stratum mucosum, not those of stratum corneum or dermis.[14] Finally, Giosué Sangiovanni in 1819 was the first to describe melanocytes in the squid, which he termed as ‘chromatophores’.[14] In 1837, Friedrich Henle also identified pigment producing cells in human epidermis, as identical to pigment cells of the eye.[14] In 1879, Moritz Kaposi was one of the first to observe lack of pigment granules in the rete pegs of vitiligo.[15] To close this chain of historical events, Bruno Bloch in 1917 described the DOPA reaction demonstrating the melanin synthesizing enzyme tyrosinase within the melanocyte.[16]

In summary, around 4000 years of known history elapsed from the time man became aware of disturbing white spots on the skin until the melanocyte was finally identified as the responsible actor for depigmentation and other pigmentary disorders.

## The Hair Follicle Unit as the Melanocyte Reservoir in Vitiligo

### Previous findings

During the first half of the twentieth century, some progress was made in the areas of pigment cell morphology and function. However after 1950 until present, intensive research provided important information on melanocyte structure, gene characterization, physiology, physiopathology and bio-molecular characteristics, although the pathogenesis of vitiligo has not been completely elucidated;[17] therefore, halting depigmentation and inducing complete repigmentation in all vitiligo patients, especially in acral, rapidly progressing and widespread vitiligo is awaiting for a definitive response.

During the second half of the twentieth century in 1959, Staricco RG showed amelanotic melanocytes in the external root sheath of hair follicles that suggested immature pigment cells.[18] Further a melanocyte reservoir was proposed in the hair follicle by Ortonne *et al*., in 1979 who described the presence of DOPA negative, non-dendritic pigment cells along the external root sheath that migrated toward the basal cell layer to become functionally active in patients treated with PUVA for vitiligo.[19] Such findings were confirmed by Cui *et al*., in 1991, when treatment in vitiligo patients stimulated inactive melanocytes in the middle and/or lower parts of hair follicles to proliferate and migrate along the outer root sheath to the nearby epidermis, where pigment cells expanded radially and were clinically observed as perifollicular repigmentation.[20] In addition, after transplantation of the lower portion of autologous pigmented scalp hair follicles into depigmented vitiligo skin, DOPA positive melanocytes were detected in the repigmented epidermis suggesting a pigment cell reservoir present in this anatomical site.[21] Later on, Grichnik *et al*., in 1996 demonstrated immature dendritic, tyrosinase negative and c-kit positive pigment cells mainly concentrated around the follicular ostium, and to a lesser extent in the rete pegs and the outer root sheath,[22] suggesting a source of not fully mature pigment cells that were available for epidermal repigmentation.

### The hair follicle structure and function

The human hair follicle is a specialized skin appendage arising from epithelial-mesenchymal interactions beginning around the third month of embryonic development with the formation of the dermal papilla; further proliferation and differentiation of more than 20 different types of cells overlying the papilla initiate the hair follicle structure with six main compartments: the connective tissue sheath, the dermal papilla fibroblasts, the outer root sheath, the inner root sheath, the shaft and the sebaceous gland.[23]

The mature hair follicle consists of a morphologically permanent upper segment and a lower segment that remodels during hair cycling in which three phases are described: (a) anagen, where the growth phase occurs generating the hair shaft, the inner and outer root sheaths and the hair matrix; (b) catagen, with epithelial regression post apoptosis; and (c) telogen, a quiescent phase with resting cells.[24,26]

In the bulbar region, multiple, dendritic, tyrosinase positive, large differentiated melanocytes located within the hair matrix provide melanin for hair shaft pigmentation. All of these anatomical structures constitute the pigmentary hair follicle unit bearing the melanocyte reservoir.

## Stem Cells and the Niche

Stem cells: Mammalian stem cells are divided in two categories: (1) Stem cells that may differentiate into all of the specialized embryonic tissues, and (2) Stem cells that are present in adult tissues and are capable of regenerating and maintain the normal tissue turnover and repair by providing new specialized and differentiated cells; these cells must be immature, slow cycling, self-maintaining and fully competent to regenerate progeny.[27–29]

Tissue-specific stem cells are usually found in a specialized environment within specific organs called the niche that provide important signals to guide their function[27] and are assumed to share two important characteristics:

- the ability for indefinite self-renewal; some stem cells remain in the niche and others become a lineage-restricted transient amplifying cell or progenitor cell that leaves the niche and undergo several limited cycles of proliferation before transforming into differentiated cells[30]; and
- the capacity to differentiate into multi specialized cell lineages of the specific tissue.[29]

### The niche and melanocyte stem cells

**Figure 4:**
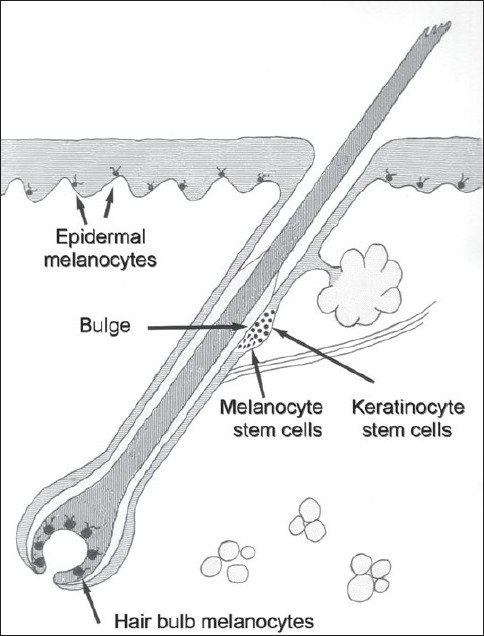
The melanocyte reservoir. Illustration depicts the location of keratinocyte and melanocyte stem cells. Other, mature terminal melanocytes are also shown

As mentioned, it is assumed that stem cells are maintained in a specific environment known as the niche. Nishimura *et al*., in 2002 identified melanocyte stem cells in the lower permanent portion of Dct-lacZ transgenic mice hair follicles, which become activated at early anagen as they are tightly coupled to the hair regeneration cycle.[31] They are located in the lower part of the hair follicle bulge, just below the hair follicle stems cells.[32] The bulge region of the hair follicle, defined as the portion of the outer root sheath of the hair follicle at the insertion site of the arrector pili muscle, constitutes currently the best characterized site of epidermal stem cell populations [Figure 4]. In addition, there is evidence of other stem cells located in the interfollicular epidermis, that is, the portion of the epidermis located between the orifices of hair follicles.[27]

Recently, when studying hair follicles from normal human scalp skin with immunohistological staining techniques and quantitative histomorphometry, the bulge region was found as a site of relative immune privilege, protecting the hair follicle epithelial stem cell reservoir from autoaggressive immune attacks,[33] a finding that would not be surprising if occurring in pigment stem cells as well and would constitute a possible explanation for the presence of pigmented hairs in vitiligo lesions.

Even though hair follicles are absent in palmar skin, its repigmentation following NB UVB or PUVA in vitiligo[34] raises the question on immature melanocyte precursors or stem cells remaining in vitiliginous lesions; in fact, a population of cells with molecular characteristics of stem cells and transient amplifying cells related to keratinocytes has been demonstrated at the tips of deep rete ridges in the interfollicular skin of breast, palms and soles;[35,37] the possibility of similar stem cells related to melanocytes residing in any area of the skin which could act as a reservoir for such areas cannot be excluded, and the possible design of future therapeutic interventions at this level should not be underestimated.

#### Melanogenesis

Repigmentation arises mostly from hair follicle units wherever hair is available, but unaffected melanocytes may be present in vitiligo areas even many years after depigmentation takes place[38] and those at the edge of lesions may also contribute to repigmentation.[4,39]

Hair pigmentation is provided by very active follicular melanocytes located above the dermal papilla.[40] Synthesis and transfer of melanin granules are regulated by a group of enzymes, structural and regulatory proteins, transporters, receptors and their ligands, acting on all development stages and cells of the hair follicle. Follicular melanogenesis is tightly coupled to the anagen stage of the hair cycle, becoming switched off during catagen and remaining absent through telogen. During each hair cycle, at the point from the anagen to catagen, melanocytes in the hair bulb matrix undergo apoptosis and their reconstitution occurs just when the next anagen phase begins, a sequence that is possible because of stem cells at the bulge that activate and induce melanocyte proliferation and migration; this cycle repeats once and again producing melanin granules for hair shaft pigmentation.[31]

The regulation of melanogenesis is modulated by melanocortin 1 receptor, adrenocorticotropic hormone, melanocyte stimulating hormone, agouti protein ligands, c-Kit, and endothelin receptors.[41] It is possible that the gradual deterioration of stem cells to remain active may be part of the cause of hair graying.[42,43]

## The Role of Proinflammatory Cytokines and UV Light

For repigmentation to occur, it is necessary that melanocytes become stimulated with appropriate signals. In this regard, two important properties of melanocytes have to be taken into consideration: (a) neo-melanogenesis, which implies melanin synthesis and production of melanosomes and b) melanocyte migration, which will help pigment cells to reach depigmented skin.

### Cytokines

During *in-vitro* stimulation of pigment cells, several cytokines have been observed to have different effects: a) PGD2, LTB4, LTC4, LTD4, LTE4, thromboxane B2 and HETE produce increased dendricity, edema, higher levels of tyrosinase and immunoreactive b-locus;[44,45] b) other cytokines including transforming growth factor alpha, basic fibroblast growth factor (bFGF), stem cell factor (SCF) and endothelin-1 (ET-1), increase melanocyte migration;[46,47] some of these cytokines (LTB4, LTC4, ET-1, EGF) are involved in both functions.

In addition, the SCF/KIT pathway plays a critical role in the control of normal human melanocyte homeostasis.[48] It is conceivable that in the future, cytokines may be used to induce melanocyte migration and stimulate repigmentation.[49]

### UV light

Both UVA and NB UVB are potent melanocyte stimulants for repigmentation; sunlight overexposure with the full UV spectrum may induce marked pigmentation with diffuse skin darkening that depends on the intensity of UV light exposure.

### Ultraviolet radiation produces two effects on vitiligo skin:

- Immunosuppression: To stop melanocyte destruction after UVB irradiation T-regulatory (suppressor) cell activity is induced and released; IL10 may be important for the differentiation and activation of T-regulatory cells having suppressor autoimmune activity.[50] A higher suppressive effect has also been observed with narrow band NB UVB on systemic immune responses.[51] In addition, sera from patients after PUVA contain higher levels of bFGF, SCF and hepatocyte growth factor, which may induce re-growth of melanocytes.[52]
- Stimulation of growth factors: these molecules may be activated with UVR; this has been shown with UVR as the number of residual melanocytes increases most probably by enhancing melanocyte growth factors such as bFGF and ET-1.[53,54]

### The Significance of Leukotrichia

The presence of pigmented hairs in vitiliginous skin is a good prognostic sign for vitiligo recovery speaking in favor of an intact melanocyte reservoir.[55] Nevertheless, after a number of years, in very active *vitiligo vulgaris* melanocytes may become gradually destroyed leading to leukotrichia, although it is not correlated with disease activity.[56]

**Figure 5:**
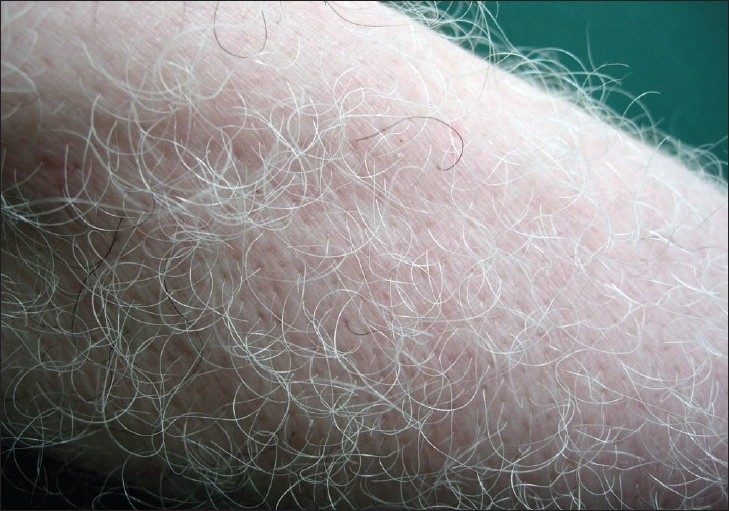
Leukotrichia. Hair pigmentation has been completely lost many years after vitiligo onset. At this point, repigmentation is not possible with medical therapy because of the complete destruction of the melanocyte reservoir

As leukotrichia represents melanocyte reservoir exhaustion [Figure 5], repigmentation of depigmented skin in vitiligo by medical therapies is very unlikely and melanocyte transplantation may be the best therapy for depigmented skin.[57]

In addition, leukotrichia may be distressing in certain anatomical areas and patients may request therapeutic advice; hair repigmentation may be accomplished as reported previously with epidermal grafts[58] and thin split thickness grafts.[59] A recent noteworthy finding with epilation therapy disclosed the development of leukotrichia in 20 patients from a group of 821 individuals treated with a noncoherent IPL system, with a 650 nm flashlamp filter who did not recover in 2-6 months, suggesting that patients with vitiligo should not have light epilation therapies.[60]

## Conclusions

Vitiligo is a complex disease producing asymptomatic depigmentation but with a heavy psychological burden. Complete knowledge of its etiology has been elusive for decades of intense research; but during the past few years important findings started to shed light on its causes and pathogenesis of depigmentation. The complete knowledge of the melanocyte reservoir is of prime importance to understand the mechanisms of repigmentation, which are crucial for designing newer strategies for vitiligo therapy. The possibility of melanocyte stem cells in skin areas without hair follicles where repigmentation occurs with a diffuse pattern may suggest the existence of an amplified concept of the melanocyte reservoir that would include the interfollicular epidermis as well.
